# Robust Regularization Enables Automated, Real-Time Square-Wave Voltammetry Signal Quantification

**DOI:** 10.64898/2026.07.25.740173

**Published:** 2026-07-27

**Authors:** Max Yates, Jenny Ji, Steven Yee, Hyongsok T. Soh

## Abstract

Square-wave voltammetry (SWV) is widely used for electrochemical biosensing because it enables sensitive, temporally resolved measurement of redox reporter signals. However, automated quantification of SWV signal remains challenging for long duration and *in vivo* measurements, where voltammograms can exhibit changing baselines, heterogeneous noise, peak drift, outliers, and interfering faradaic processes. Here, we introduce the Adaptive Square-Wave Voltammetry Iterative Fitting Toolkit (ASWIFT), an automated method for robust SWV signal extraction based on iteratively reweighted regularized smoothing. ASWIFT is available as both an open-source Python package and a downloadable desktop application. The method adaptively estimates the baseline, selects regularization strengths, fits the redox peak, and reports peak height without trace-specific parameter tuning. Across simulated datasets spanning diverse baseline, peak, noise, and concentration-response conditions, ASWIFT produced less systematic bias and more consistent signal estimates than existing methods. We further evaluated ASWIFT using *in vitro* doxorubicin measurements and *in vivo* DNA-based kanamycin sensor measurements collected in rat blood and interstitial fluid, demonstrating agreement with established methods. These results support ASWIFT as a robust framework for automated SWV analysis in real-time electrochemical biosensing.

## Full Text Availability

The license terms selected by the author(s) for this preprint version do not permit archiving in PMC. The full text is available from the preprint server.

